# The association between fruit and vegetable intake and depression: evidence from genetic studies

**DOI:** 10.1016/j.jnha.2024.100332

**Published:** 2024-08-10

**Authors:** Yifan Zhang, Ruijun Chen, Yuexin Yan, Shengyuan Su

**Affiliations:** Department of Intensive Care Medicine, ShenzhenBaoan District People’s Hospital, Shenzhen, China

To the Editor:

We are very interested in reading the study by Huiqi Li et al. [1] titled "Singapore Chinese Health Study: Association Between Midlife Fruit and Vegetable Consumption and Late-Life Depressive Symptoms," which analyzed 13,738 Singaporean adults. The study found a negative association between fruit intake in midlife and the incidence of depressive symptoms, especially with higher intake of oranges, tangerine, bananas, papayas, and watermelons significantly lowering the occurrence of depressive symptoms. Vegetable consumption showed no significant association with the incidence of depressive symptoms. This finding holds significant implications for public health policy, suggesting increased fruit intake in midlife to improve mental health in older adults. We commend the authors' work but also note a few minor issues that need to be addressed.

Although the study has considered various potential confounding factors such as age, gender, education level, marital status, body mass index, smoking, alcohol consumption, red meat intake, total energy intake, physical activity, sleep duration, and underlying diseases, there may still be uncontrolled confounding factors. For instance, participants' mental health history and family support systems could potentially influence the results. The study did not conduct detailed subgroup analyses for different subgroups (e.g., different genders, age groups, socioeconomic statuses), which could obscure important findings within specific populations. Additionally, the study excluded participants with cognitive impairment and diagnosed depression, and did not analyze genetic susceptibility to depression. Therefore, we performed a two-sample Mendelian randomization (MR) study on the association between fruit consumption and depression. We utilized single nucleotide polymorphisms (SNPs) associated with fruit intake, orange intake, banana intake, and vegetables from genome-wide association studies (GWAS) as instrumental variables (IVs). The outcome variables included GWAS data on depression from the UK Biobank databases. We combined ratio estimates using the inverse-variance weighted (IVW) method and the weighted median method, applying false discovery rate (FDR) correction to the P-values. Additionally, we performed MR-Egger regression and MR-PRESSO tests to assess for pleiotropy.

Our study results demonstrate that, based on the IVW method and fixed-effects models, genetic predictions indicate that increased intake of fresh fruits significantly reduces the risk of depression (adjusted P < 0.001, OR 95% CI: 0.939 [0.918, 0.959]). Additionally, intake of oranges and bananas is associated with reduced risk of depression (adjusted P < 0.001, orange OR 95% CI: 0.966 [0.954, 0.978]; banana OR 95% CI: 0.971 [0.968, 0.975]). Moreover, consumption of vegetables is also linked to lower incidence of depression (adjusted P < 0.001, OR 95% CI: 0.974 [0.967, 0.982]). Salad and raw vegetable intake are considered protective against depression (adjusted P < 0.001, OR 95% CI: 0.942 [0.928, 0.955]). The study also found that adding vegetables to homemade soups reduces depression risk (adjusted P < 0.001, OR 95% CI: 0.931 [0.898, 0.965]). However, other forms of vegetable intake (adjusted P < 0.001, OR 95% CI: 1.034 [1.026, 1.042]) or pure vegetables/vegetable juices intake (adjusted P = 0.017, OR 95% CI: 1.009 [1.003, 1.015]) may increase the risk of depression. Conversely, canned vegetables, vegetables in canned soups, and mixed vegetables showed no significant impact on depression incidence (see Table 1).

**Table 1:** MR analysis results assessing the potential causal relationships between fruit and vegetable intake and the risk of depression using the Inverse-Variance Weighted (IVW) method, after correcting *P-*value using the False Discovery Rate (FDR) method.

Exposure	Outcomes	Method	Beta	Se	*P*_value	*P_*adjust	OR (95%CI)
Fresh fruit intake	Depression	IVW	−0.063	0.011	<0.001*	<0.001*	0.939 (0.918, 0.959)
Orange intake	Depression	IVW	−0.035	0.006	<0.001*	<0.001*	0.966 (0.954, 0.978)
Banana intake	Depression	IVW	−0.030	0.001	<0.001*	<0.001*	0.971 (0.968, 0.975)
Vegetable consumers	Depression	IVW	−0.026	0.004	<0.001*	<0.001*	0.974 (0.967, 0.982)
Cooked vegetable intake	Depression	IVW	0.007	0.017	0.683	0.853	1.007 (0.974, 1.041)
Salad/raw vegetable intake	Depression	IVW	−0.060	0.007	<0.001*	<0.001*	0.942 (0.928, 0.955)
Ingredients in canned soup: Vegetables	Depression	IVW	0.038	0.031	0.214	0.534	1.039 (0.978, 1.103)
Ingredients in homemade soup: Vegetables	Depression	IVW	−0.072	0.019	<0.001*	<0.001*	0.931 (0.898, 0.965)
Mixed vegetable intake	Depression	IVW	0.001	0.002	0.705	0.705	1.001 (0.997, 1.005)
Other vegetables intake	Depression	IVW	0.033	0.004	<0.001*	<0.001*	1.034 (1.026, 1.042)
Pure fruit/vegetable juice intake	Depression	IVW	0.009	0.003	0.003*	0.017*	1.009 (1.003, 1.015)

An asterisk (*) signifies a statistical difference, implying a causal relationship between the two variables.

Our MR study enriches the understanding of the causal relationship between fruit and vegetable intake and depression. Compared to Huiqi Li's research, we identified several similarities and differences: (1) Both studies support the beneficial effects of fruit intake on depression, especially oranges and bananas. (2) While Li's study did not find an association between vegetable intake and depression, our research suggests that vegetable consumption can reduce depression risk, particularly when salads or vegetables are added to homemade soups, showing a significant negative association with depression. However, pure vegetables or vegetables with high carbohydrate content may increase the risk of depression, factors that may not have been considered in Li's study. (3) Our study focused on a European population, whereas Li's study focused on an Asian population, highlighting a difference between the two studies. (4) Our study employed genetic polymorphism analysis methods, while Li's study used a prospective cohort study design, potentially providing higher-level evidence. Mechanistically, naturally occurring folate and B-complex vitamins in foods such as fruits and vegetables may be particularly beneficial for preventing depression [2,3], while the high concentration of antioxidants in fruits and vegetables is also strongly associated with mental health [4]. However, this study is still subject to several limitations. The MR assumes that the effects of SNPs on the exposure (fruit and vegetable intake) are independent of their effects on the outcome (depression), which may not be the case in this instance. For example, certain SNPs may influence both diet and depression by shaping personality traits. Additionally, the impact of depression may also stem from other dietary aspects that are correlated with it. This study did not uncover a association between vegetables and fruits specifically, and as an MR study, it was unable to determine their individual contributions within the overall diet.

Our findings, along with those of Li's study, exhibit conflicting effects of different types of fruits and vegetables on specific health outcomes such as depression, potentially due to multiple factors. Firstly, these discrepancies could reflect variations in research methodologies, heterogeneity among population samples, and limitations in study designs. Different studies may employ distinct definitions, classifications, or methods to assess fruit and vegetable intake, leading to inconsistent results. Secondly, the diversity of individual dietary habits significantly contributes to the contradictory findings. Varying dietary patterns across regions and cultural backgrounds can result in different intakes and types of fruits and vegetables, thereby influencing the observed health effects in diverse populations. It is crucial to emphasize that compared to Li's study, the effect sizes of fruits and vegetables on depression in our study are minuscule, often indicative of the interplay between immense statistical power, as in the UK Biobank, and weak residual confounding.

Overall, we highly appreciate the findings of Li et al.'s study. Nevertheless, we are far from concluding that fruit consumption can definitively prevent depression. Given the complexity and diversity of factors influencing depression, including socioeconomic status and educational level, which may differentially impact depression risk, further investigations across various countries and research designs are imperative to explore the association between depression and fruit and vegetable consumption.

## Authors’ contributions

YifanZhang acquired the data and performed the main MR analyses and drafted the manuscript. RuijunChen and YuexinYan: Supervision, ShenyuanSu: review & editing.

## Financial

The authors received no financial support to produce this manuscript.

## Conflict of interest

The authors declare that they have no competing interests.

## Data availability statement

All GWAS summary statistics can be downloaded from GWAS catalog.
